# Antimicrobial activity of thiophene derivatives derived from ethyl (E)-5-(3-(dimethylamino)acryloyl)-4-methyl-2-(phenylamino)thiophene-3-carboxylate

**DOI:** 10.1186/s13065-017-0307-z

**Published:** 2017-08-03

**Authors:** Yahia Nasser Mabkhot, Nahed Ahmed Kaal, Seham Alterary, Salem S. Al-Showiman, Thoraya A. Farghaly, Mohammad S. Mubarak

**Affiliations:** 1 Department of Chemistry, College of Science, King Saud University, P.O. Box 2455, Riyadh, 11451 Saudi Arabia; 2 Chemistry Department, Faculty of Science, Cairo University, Giza, 12613 Egypt; 3 Department of Chemistry, Faculty of Applied Science, Umm Al-Qura University Makkah Almukkarramah, Mecca, 21514 Saudi Arabia; 4 Departmentof Chemistry, The University of Jordan, Amman, 11942 Jordan

**Keywords:** Enaminones, Heteroamines, Antimicrobial activity, Heterocycles

## Abstract

**Background:**

The thiophene nucleus has been recognized as an important entity in the synthesis of heterocyclic compounds with promising pharmacological characteristics.

**Results:**

A number of new heterocyclic compounds incorporating thiophene species have been prepared from the titled enaminone via the reaction with different nucleophiles and electrophiles. The structure elucidation of the designed compounds was derived from their spectral information. The results of antimicrobial activity of the prepared compounds revealed that derivatives **7b** and **8** exhibited activity comparable to the standard drugs ampicillin and gentamicin for all tested bacteria species. Additionally, compound **3** displayed potent activity against *Aspergillus fumigates*, whereas compounds **5**, **6**, and **7a** showed good activity against *Syncephalastrum racemosum*.

**Conclusions:**

We have synthesized a number of new thiophene-containing compounds. The results of antimicrobial activity of the prepared compounds revealed that changing the substituents at position-2 of thiophene ring significantly affect their biological activity. The pyridine side chain derivatives in compounds **7a**, **7b** and **8** showed excellent antimicrobial activity.

## Background

**Chart 1:**
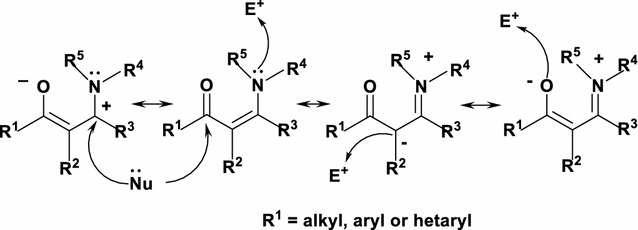
The nucleophile and electrophilic sites of enaminones

Enaminones have been proved to be extremely stable species and form a versatile class of valuable precursors for the preparation of sundry classes of organic compounds [1–4]. Their reactivity is referred to the actuality that they consolidate the ambident nucleophilicity of enamines and electrophilicity of enones. For example, each enaminone can be attacked by a given nucleophile at the two sites, C-3 (the dialkylaminoethylene group) and C-1 (the carbonyl group) with the reactivity order C-3 > C-1. In addition, it can be attacked by an electrophile at C-2, oxygen and/or nitrogen sites with reactivity order C-2 > N > O (Chart 1).

On the other hand, the thiophene nucleus has been recognized as an important entity in the synthesis of heterocyclic compounds with promising pharmacological characteristics. An extensive variety of therapeutic applications of thiophene derivatives has been surveyed in the literature [5–8]. Thiophene moiety and their derivatives are known as diabetes mellitus [9], antihypertensive [10], antimicrobial [11], analgesic and anti-inflammatory [12], cholesterol inhibitors [13], antiviral [14], and antitumor agents [15]. Encouraged by all these findings and all the promising biological results we obtained in our laboratory [16–24], we report, herein, an efficient and rapid method for the synthesis of a series of thiophene derivatives from the titled enaminone and investigated their antimicrobial activity. Such a study depends on the change the substituent at position-2 of the thiophene ring to investigate their effect on the activity against the various microbial species used. Also, based on the results obtained in our laboratory and recently published [11] from the preparation of thiophene compounds and gave good results as antimicrobials, we preferred the preparation of new thiophene compounds by substituting the phenyl group by methyl one on the thiophene loop to investigate the improvement of their biological outcome of synthesized compounds.

## Results and discussion

### Synthesis

**Scheme 1:**
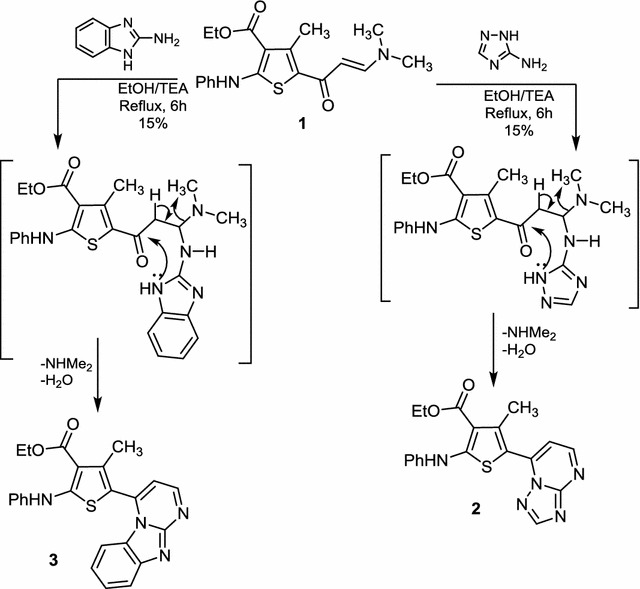
Reaction of enaminones with heteroamines

Enaminone **1**, required in this investigation was prepared according to published procedures [25]. Compound **1** was reacted with two nitrogen nucleophiles namely, 3-aminotriazole and 2-aminobenzimidazole in ethanol in the presence of triethylamine and ZnCl_2_ to afford the fused pyrimidine derivatives **2** and **3**, respectively (Scheme 1). Compounds **2** and **3** were characterized by a panel of spectroscopic techniques and by elemental analysis. IR spectra of **2** and **3** revealed the disappearance of the ketonic carbonyl group present in the enaminone **1** and the appearance of carbonyl groups of acetyl or ester groups, respectively. ^1^H-NMR spectrum of compound **3** in DMSO-*d*_*6*_ showed a triplet (*J* = 6.0 Hz) and a quartet (*J* = 6.0 Hz) at δ 1.36, 4.32 ppm, due to the methyl and methylene hydrogens of the ester group, respectively. The methyl group attached to the thiophene ring appeared as a singlet at δ 2.62 ppm, whereas the pyrimidine protons appeared as doublets (*J* = 4.5 Hz) at δ 6.32 and 7.58 ppm. Aromatic protons resonated as a multiplet at 6.89–7.60 ppm while the NH proton appeared as a singlet at 10.13 ppm. Such results indicate that the mechanism of the latter reaction proceeded via nucleophilic attack of the exocyclic amino group of triazole at the activated double bond of enaminone **1** to afford the Michael-type intermediate, which underwent intramolecular cyclization with concurrent elimination of NHMe_2_ and H_2_O molecules to give the final products **2** or **3**, as illustrated in Scheme 1.

**Scheme 2:**
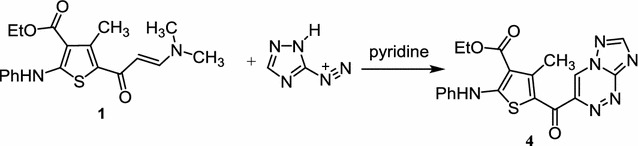
Synthesis of compound **4**

On the other hand, enaminone **1** was coupled with the diazonium salt of 3-amino-1,2,4-triazole in pyridine to yield ethyl 5-([1, 2, 4] triazolo[5,1-c] [1, 2, 4] triazine-3-carbonyl)-4-methyl-2-(phenylamino)-thiophene-3-carboxylate (**4**) (Scheme 2). Its mass spectrum showed a molecular ion peak at *m/z* 408 (M^+^) and the ^1^H NMR spectrum of such compound indicated the presence of singlet signals at δ 8.52 and 8.76 ppm assigned to the =CH of triazine and triazole rings. Additionally, ^13^C NMR of compound **4** revealed signals for all 17 carbons.

**Scheme 3:**
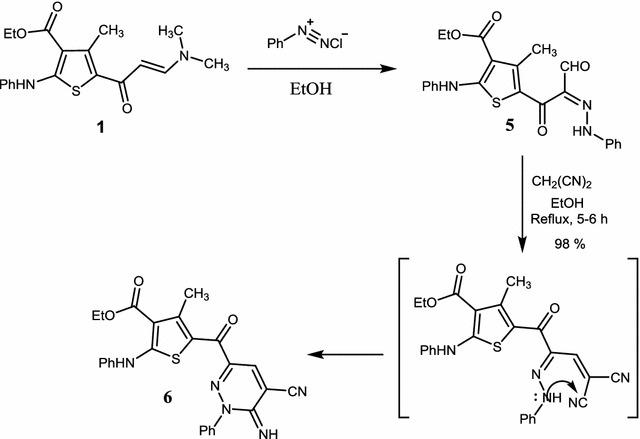
Synthesis of compounds **5** and **6**

Syntheses of compounds **5** and **6** were achieved by coupling of enamenone **1** and benzenediazoniumchloride in ethanol. The solid products were filtered and recrystallized from ethanol to afford the desired compounds in pure forms. Reaction of compound **5** with malonitrile in ethanol, under reflux, afforded compound **6** [26, 27] (Scheme 3). Structures of these two compounds were confirmed by spectroscopic methods. IR spectrum of **5** showed absorption bands at 1594 and 1650 cm^−1^ due to C=N, and C=O stretching, respectively. In addition, absorption bands attributed to the carbonyl group of the ester and to the NH stretching appeared at 1706 and 3450 cm^−1^, respectively. In the ^1^H-NMR spectrum of compound **5**, protons of the two phenyl groups appeared as a multiplet in the range 7.00–7.30 ppm, whereas protons of the two NH groups appeared as singlets at δ 9.94 and 10.37 ppm. The aldehydic hydrogen appeared as a singlet at δ 14.34 ppm and protons of the ester group appeared as a triplet and a quartet (*J* = 6.0 Hz) at δ 1.36, 4.32 ppm, respectively, whereas the methyl group attached to the thiophene ring appeared as a singlet at δ 2.67 ppm. The ^13^C-NMR spectrum was also consistent with the assigned structure and the following signals were observed: δ 13.9 (CH_2_CH_3_), 17.1 (CH_3_), 60.2 (CH_2_CH_3_), 112.5 (C=N), 108.8, 116.3 (2C), 119.8, 120.0 (2C), 124.2, 125.6, 129.2 (2C), 129.2 (2C), 132.5, 140.7, 150.0, 164.2 (Ar–C), 167.0 (C=O) for ester, 180.9 (C=O), 189.0 (C=O) for aldehyde. On the other hand, the mass spectrum of compound **5** displayed the molecular ion [M]^+^ (100%) at *m/z* = 435 corresponding to the molecular formula C_23_H_21_N_3_O_4_S.

For compound **6**, the IR spectrum displayed absorption bands at 1706 and 1595 cm^−1^ attributed to the two carbonyl groups. An absorption band due to C=N stretching appeared at 1551 cm^−1^ and the cyanide CN band appeared at 2197 cm^−1^. In addition, two bands appeared at 3447 and 3366 cm^−1^ due to the two NH bonds. The ^1^H-NMR spectrum of compound **6** showed a triplet at δ 1.25 ppm (*J* = 6.0 Hz) due to the methyl protons of the ester group, whereas the other methyl group appeared as a singlet at δ 2.67. The methylene protons of the ester group appeared as a quartet at δ 4.21 ppm (*J* = 6.0 Hz). The imine proton (=NH) proton appeared as a singlet at δ 8.10 ppm and the pyridazine proton appeared also as singlet at δ 8.49 ppm. Another singlet at δ 10.66 ppm due to the NH protn appeared in the spectrum. The aromatic protons of the phenyl group appeared as a multiplet in the range δ 7.19–7.61 ppm. In the ^13^C-NMR spectrum the following signals were observed: δ 14 (CH_2_CH_3_), 17.75 (CH_3_), 51.74 (CH_2_CH_3_), 120.78, 121.01, 124.83, 125.83, 126.21, 129.04, 129.12, 164.01 (Ar–C).

**Scheme 4:**
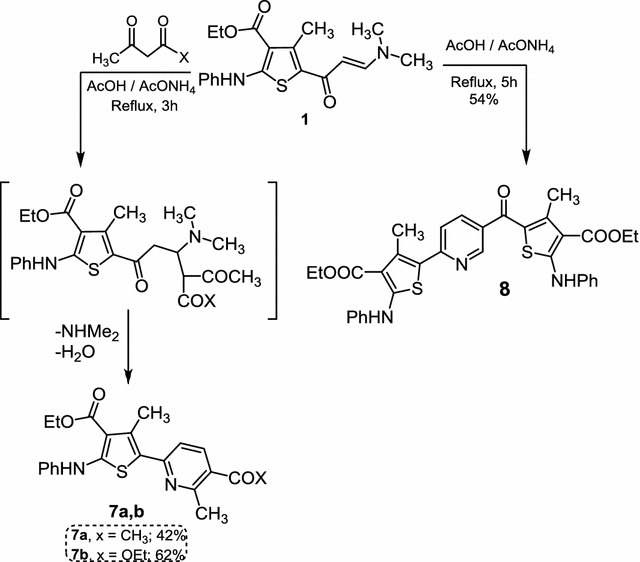
Synthesis of compounds **7a**,**b** and **8**

Next, reactivity of enaminone **1** was investigated towards C-nucleophiles. Reaction of enaminone **1** with active methylene compounds in acetic acid in the presence of ammonium acetate led to formation of compounds **7a**,**b** (Scheme 4). The reaction may proceed by an initial Michael addition of the active methylene compound to the activated double bond of **1** followed by a tandem elimination of NHMe_2_ and condensation with ammonia to give compounds **7a**,**b**. Structures of pyridine derivatives **7a**,**b** was established on the bases of spectral data (see “Experimental section”). Heating enaminone **1** in acetic acid and in presence of ammonium acetate gave 5-(6-(4-(Ethoxycarbonyl)-3-methyl-5-(phenylamino)thiophen-2-yl) nicotinoyl)-4-methyl-2-(phenylamino)-thiophene-3-carboxylic acid Ethyl ester (**8**) (Scheme 4). Based on its ^1^H NMR and Mass spectra, its structure was proved as illustrated in experimental part.

**Scheme 5:**
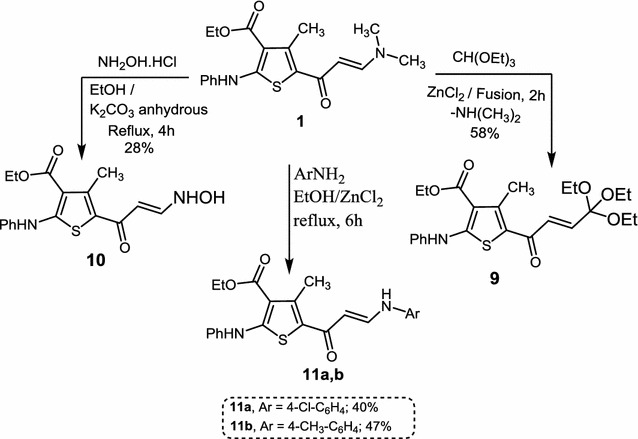
Preparation of compounds **9**–**11**

Compound **9** was synthesized via melting the enamenone **1** with triehylorthoformate (TEOF) in presence of zinc chloride as a catalyst, (Scheme 5) followed by addition of ethanol; the precipitate was filtered to afford the desired product as pure crystals. In the ^1^H-NMR spectrum of compound **9** two triplets appeared at δ 1.39 and 1.40 ppm attributed to the two methyl groups of the ether and ester, respectively. In addition, a singlet at δ 2.17 due to the methyl group that is attached to thiophene ring also appeared. The two methylene groups (CH_2_) of the ester and ether appeared as quartets at δ 4.36 and 4.35, respectively. On the other hand, the vinylic proton (CH=CH) appeared as two doublets at δ 5.59 and 7.69 (*J* = 12.0 Hz), whereas the aromatic protons appeared as a multiplet in the range δ 7.10–7.51 ppm and the NH proton as a singlet at δ 10.51 ppm. ^13^C-NMR is in total agreement with the assigned structure. The different carbon atoms appeared at the following δ: 14.4 (CH_2_CH_3_), 16.9 (CH_3_), 60.3 (CH_2_CH_3_), 95.2, 153.0 (CH=CH), 109.7, 119.7, 122.4, 123.8, 129.5, 140.3, 141.5, 160.7 (Ar–C), 167.2 (C=O), 182.3 (C=O).

The mass spectrum of compound **9** displayed the molecular ion [M]^+^ at *m/z* = 461 (6%), corresponding to the molecular formula (C_24_H_31_NO_6_S). Fragments at 446 [M-15]^+^ (36%), 306 [M-155]^+^ (100%), among others also appeared.

Compounds **10** and **11a**,**b**, were prepared by refluxing a mixture of compound **1** and hydroxylamine hydrochloride or aniline derivatives in ethanol in the presence of anhydrous K_2_CO_3_ or ZnCl_2_ as a catalyst. IR spectrum of the prepared compound **10** showed absorption bands at 3427 cm^−1^ due to NH and OH groups, and bands at 1705 and 1655 cm^−1^ attributed to the two carbonyl groups [28]. ^1^H-NMR spectrum in CDCl_3_ of compound **11a** displayed two signals (appeared as singlets) for two NH protons at δ10.03 and 10.14 ppm.

### Biological screening

#### Antibacterial and antifungal activity of prepared compounds

All synthesized compounds were screened for their antibacterial (Gram-positive and Gram-negative) and antifungal activities at a concentration of 5 mg/mL. Ampicillin, gentamycin, and amphotericin ***B***, were employed as standard antibacterial agents (Gram-positive and Gram-negative) and antifungal, respectively. The tested fungi were *A*. *fumigates*, *S*. *racemosum*, *Geotrichum candidum*, and *Candida albicans*. Tested Gram-positive bacteria were *Streptococcus pneumoniae* and *Bacillus subtilis*, whereas Gram-negative were *Pseudomonas aeruginosa* and *Escherichia coli* Susceptibilities of microbial isolates to the test compounds were evaluated by measuring the average diameter of inhibition zones of bacterial growth surrounding the well (in millimetres) compared to the reference drugs. The obtained results reflected variable antimicrobial activity. Among the test compounds, derivatives **9b** and **10** were the most potent against all tested fungi species with a 100% inhibition zone which is similar to *amphotericin B* as a reference standard. Compounds **3**, and **10** showed good potency against *Aspergillus fumigatus* (78.9 and 73% inhibition zone, respectively). Furthermore, derivatives **5**, **6**, **7a**, and **3** were the most potent derivatives with 95.5, 88.3, 87.3 and 85.8% inhibition zones, respectively, against *S*. *racemosum*. The other thiophene derivative **7a** displayed high potent activity of 85.7% inhibirion zone against *Geotricum candidum*. The rest of prepared compounds exhibited moderate to mild activity as illustrated in Table 2.

Significant activity was observed for some of the test compounds, such as **7b** and **8**, against all Gram-positive and Gram-negative bacteria. Compound **9a** exerted potency of 102 and 98.8% inhibition zone, respectively compared to gentamicin as a reference standard against Gram-negative bacteria (Table 1).

**Table 1:** Antibacterial activity of synthesized compounds (zone of inhibition in diameter in mm)

Tested microorganisms comp. no	Gram positive bacteria inhibition zone diameter in mm and (%) value		Gram negative bacteria inhibition zone diameter in mm and (%) value	
	*Streptococcus pneumoniae*	*Bacillus subtilis*	*Pseudomonas aeruginosa*	*Escherichia coli*
St.	Ampicillin		Gentamicin	
	23.8 ± 0.2	32.4 ± 0.3	17.3 ± 0.1	19.9 ± 0.3
**2**	16.9 ± 0.58 (71.0%)	18.2 ± 0.44 (56.2%)	10.7 ± 0.34 (61.8%)	11.9 ± 0.63 (59.8%)
**3**	12.9 ± 0.63 (54.2%)	13.2 ± 0.58 (40.7%)	11.8 ± 0.36 (67.2%)	10.8 ± 0.44 (54.3%)
**4**	16.7 ± 0.54 (70.2%)	17.4 ± 0.63 (53.7%)	14.1 ± 0.52 (81.5%)	13.2 ± 0.47 (66.3%)
**5**	19.3 ± 0.67 (81.1%)	14.6 ± 0.57 (45.1%)	13.6 ± 0.42 (78.6%)	14.5 ± 0.54 (72.9%)
**6**	16.5 ± 0.78 (69.3%)	17.7 ± 0.63 (54.6%)	14.2 ± 0.56 (82.1%)	15.7 ± 0.52 (78.9%)
**7a**	18.2 ± 0.44 (76.5%)	20.3 ± 0.35 (62.7%)	17.1 ± 0.34 (98.8%)	20.3 ± 0.29 (102%)
**7b**	23.8 ± 0.2 (100%)	32.4 ± 0.3 (100%)	17.3 ± 0.1 (100%)	19.9 ± 0.3 (100%)
**8**	23.8 ± 0.2 (100%)	32.4 ± 0.3 (100%)	17.3 ± 0.1 (100%)	19.9 ± 0.3 (100%)
**9**	16.4 ± 0.52 (68.9%)	13.9 ± 0.39 (42.9%)	13.2 ± 0.38 (76.3%)	12.8 ± 0.38 (64.3%)
**10**	12.8 ± 0.34 (53.8%)	15.4 ± 0.53 (47.5%)	11.9 ± 0.32 (67.8%)	11.6 ± 0.35 (58.3%)
**11a**	14.6 ± 0.58 (61.3%)	14.3 ± 0.58 (44.1%)	10.2 ± 0.32 (59.0%)	9.4 ± 0.44 (47.2%)

### Structure activity relationship (SAR)

The main objective of this study is to investigate the effect of changing the substituent at position-2 of the thiophene ring on the activity of against the various microbial species. Thus, the structure variability was only targeted in side chain groups. Observed activity reflected the importance of heterocycle side chain. Upon changing enaminone group in compound **1** into a pyridine side chain derivatives in compounds **7a**, **7b** and **8**, the antimicrobial activity was highly improved.

## Conclusions

In summary, we have synthesized a number of new thiophene-containing compounds. The newly synthesized compounds were characterized by means of a number of spectroscopic techniques and by elemental analysis. The prepared compounds were tested in vitro for their antibacterial and antifungal activity. Results revealed that changing the substituents at position-2 of thiophene ring significantly affect their biological activity. The pyridine side chain derivatives in compounds **7a**, **7b** and **8** showed excellent antimicrobial activity.

### Experimental section

#### General experimental procedures

All chemicals used were obtained from commercial sources, including Sigma-Aldrich (St. Louis, MO, USA), and were used as received without further purification, unless otherwise stated. Melting points were measured on a Gallenkamp melting point apparatus (Thermo Fisher Scientific, Paisley, UK) in open glass capillaries and are uncorrected. Infrared spectra (IR) were recorded using the KBr disc technique on a Perkin Elmer FT-IR spectrophotometer 1000 (PerkinElmer, Waltham, MA, USA). ^1^H- and ^13^C-NMR spectra were obtained with either a JEOL ECP 600 NMR spectrometer (Tokyo, Japan) operating at 600 MHz z in deuterated chloroform (CDCl_3_) or dimethyl sulfoxide (DMSO-d_6_). Chemical shifts are expressed in *δ* units and *J*-coupling constants are given in Hz. Mass spectra were acquired with the aid of a Shimadzu GCMS-QP 1000 EXmass spectrometer (Tokyo, Japan) at 70 eV. Elemental analysis was carried out on a Perkin Elmer 2400 elemental analyzer; CHN mode. Biological evaluations of the products were carried out at the medical mycology laboratory of the regional center for mycology and biotechnology of Al-Azhar University, Cairo, Egypt.

##### Synthesis of compound **2** and **3**

To a solution of compound **1** (0.358 g, 1 mmol) in absolute ethanol (15 mL) was added 3-amino-*1H*-1,2,4-triazoleor 2-aminobenzimidazole (1 mmol) in presence of two drops of triethyl amine and zinc chloride (0.2 g) as a catalyst. The mixture was heated to boiling under reflux for 6 h. the solid product was filtered while hot to afford the desired products in pure form.

##### Ethyl 5-([1, 2, 4] triazolo[1,5-a]pyrimidin-7-yl)-4-methyl-2-(phenylamino)thiophene-3-carboxylate (**2**)

Deep yellow powder in 45% yield, mp > 300 °C. IR (KBr, cm^−1^) ν_max_ = 3430 (NH), 1628 (C=O), 1547 (C=N). ^1^H NMR spectrum was not recorded due to insolubility in common solvents. MS (EIMS) *m/z*: 379 (M^+^, 95), 320 (10), 237 (100), 204 (25), 190 (12), 172 (10), 128 (10), 84 (7). Anal. Calcd. for C_19_H_17_N_5_O_2_S (379.44); C, 60.14; H, 4.52; N, 18.46. Found: C, 60.02; H, 4.34; N, 18.28%.

##### Ethyl 5-(benzo [4, 5] imidazo[1,2-a]pyrimidin-4-yl)-4-methyl-2-(phenylamino)thiophene-3-carboxylate (**3**)

Deep yellow powder, yield (25%); mp > 300 °C. IR (KBr, cm^−1^) ν_max_ = 3328 (NH), 1632 (C=O), 1553 (C=N). ^1^H-NMR (600 MHz, DMSO-*d*_*6*_)*δ* (ppm): 1.36 (t, 3H, *J* = 6.1 Hz, CH_2_CH_3_), 2.62 (s, 3H, CH_3_), 4.32 (q, 2H, *J* = 6.1 Hz, CH_2_CH_3_), 6.32 (d, 1H, *J* = 4.5 Hz), 6.89–7.60 (m, 10H, Ar–H), 10.13 (s, 1H, NH–Ph). MS (EIMS) *m/z*: 427 (M^+^, 30), 280 (20), 144 (25), 120 (100), 92 (60), 65 (23). Anal. Calcd. for C_24_H_20_N_4_O_2_S (428.51); C, 67.27; H, 4.70; N, 13.08. Found: C, 67.20; H, 4.91; N, 13.23%.

##### Ethyl 5-([1, 2, 4] triazolo[5,1-c] [1, 2, 4] triazine-3-carbonyl)-4-methyl-2-(phenylamino)-thiophene-3-carboxylate (**4**)

This compound was prepared by dissolving enaminone **1** (0.358 g, 1.0 mmol) in pyridine (10 mL) with continuous stirring at 5–10 °C. Then 1*H*- [1, 2, 4] triazole-5-diazonium nitrate, prepared from reaction of 3-amino- 1*H*-1,2,4-triazol (0.084 g, 1 mmol) with conc. HNO_3_ (1 mL) in an ice bath, was added drop-wise with stirring at 5 °C. Stirring was continued for 1 h and the mixture was allowed to warm up to room temperature. The mixture was stirred for 2 more hours. The solid was filtered, washed with water, and recrystallized from 1-butanol to afford the desired product, as pale brown needles, in 99% yield. Mp 140–142 °C.IR (KBr, cm^−1^) ν_max_ = 3260 (NH),1658 (C=O), 1593 (C=N),. ^1^H-NMR (CDCl_3_) *δ* (ppm): 1.44 (t, 3H, *J* = 6.1 Hz, CH_2_CH_3_), 2.61 (s, 3H, CH_3_), 4.42 (q, 2H, *J* = 6.1 Hz, CH_2_CH_3_), 7.08–7.43 (m, 5H, Ar–H), 8.52 (s, 1H, HC=), 8.76 (s, 1H, HC=), 10.57 (s, 1H, NH–Ph).^13^C-NMR (CDCl_3_) *δ* (ppm): 14.4 (CH_2_ CH_3_), 18.3 (CH_3_), 60.8 (CH_2_CH_3_), 106.6, 109.0, 110.3, 120.2 (2C), 124.8, 129.8 (2C), 139.8, 142.8, 143.6, 153.4, 155.2, 156.1 (Ar–C), 163.5 (C=O for ester), 166.6 (C=O). MS (EIMS) *m/z*: 408 (M^+^, 60), 288 (30), 257 (25), 213 (40), 194 (100), 165 (45), 77 (45). Anal. Calcd. for C_19_H_16_N_6_O_3_S (408.44): C, 55.87; H, 3.95; N, 20.58. Found: C, 55.64; H, 3.76; N, 20.42%.

##### Ethyl 4-methyl-5-(3-oxo-2-(2-phenylhydrazono)propanoyl)-2-(phenylamino)thiophene-3-carboxylate (**5**)

The title compound was prepared according to the following procedure: In a 100–mL Erlenmeyer flask, the diazonium salt benzenediazonium chloride, prepared from aniline (0.0279 mL), hydrochloric acid, and sodium nitrite (0.5 g in water), was added drop-wise to a solution of compound **1** in absolute ethanol (15 mL) at 0 °C. The mixture was stirred for 2 h and then left in a refrigerator for 2 more hours. The solid product was collected and recrystallized from ethanol to afford the desired product as a reddish brown powder. Yield (31%); mp > 300 °C.IR (KBr, cm^−1^) ν_max_ = 3450 (br. NH), 1706 (C=O), 1650 (C=O), 1594 (C=N). ^1^H-NMR (600 MHz, CDCl_3_) *δ* (ppm): 1.25 (t, 3H, *J* = 6.1 Hz, CH_2_CH_3_), 2.67 (s, 3H, CH_3_), 4.21 (q, 2H, *J* = 6.1 Hz, CH_2_CH_3_), 7.00–7.32 (m, 10H, Ar–H), 9.94 (s, 1H, NH–Ph) 10.37 (s, 1H, NH–Ph), 14.34 (s, 1H, CHO). ^13^C-NMR (150 MHz, CDCl_3_) *δ* (ppm): 13.9 (CH_2_CH_3_), 17.1 (CH_3_), 60.2 (CH_2_CH_3_), 112.5 (C=N), 108.8, 116.3 (2C), 119.8, 120.0 (2C), 124.2, 125.6, 129.2 (2C), 129.2 (2C), 132.5, 140.7, 150.0, 164.2 (Ar–C), 167.0 (C=O) for ester, 180.9 (C=O), 189.0 (C=O for aldehyde).MS (EIMS) *m/z*: 435 (M^+^, 100), 268 (25), 239 (20), 186 (28), 173 (21), 147 (22), 121 (10), 83 (10). Anal. Calcd. for C_23_H_21_N_3_O_4_S (435.50): C, 63.43; H, 4.86; N, 9.65. Found: C, 63.53; H, 4.80; N, 9.41.

##### Ethyl 5-(5-cyano-6-imino-1-phenyl-1,6-dihydropyridazine-3-carbonyl)-4-methyl-2-(phenylamino)thiophene-3-carboxylate(**6**)

This compound was prepared by condensation of compound **5** (0.211 g, 1 mmol) with malonitrile (0.033 g, 0.5 mmol) in ethanol (10 mL). The mixture was heated to boiling under reflux for 6 h and the solid product was collected by filtration of hot mixture to afford the desired product as a deep green powder. Yield (98%); mp > 300 °C. IR (KBr, cm^−1^) ν_max_ = 3449, 3366 (NH), 2197 (CN), 1706 (C=O), 1595 (C=O), 1551 (C=N). ^1^H-NMR (600 MHz, CDCl_3_) *δ* (ppm): 1.25 (t, 3H, *J* = 6.1 Hz, CH_2_CH_3_), 2.67 (s, 3H, CH_3_), 4.21 (q, 2H, *J* = 6.1 Hz, CH_2_CH_3_), 7.19–7.61 (m, 10H, Ar–H), 8.01 (s, 1H, =N–H), 8.49 (s, 1H, pyridazine-H), 10.66 (s, 1H, NH–Ph). MS (EIMS) *m/z*: 483 (M^+^, 80), 445 (100), 399 (28), 272 (30), 327 (45), 255 (15), 209 (12), 181 (10). Anal. Calcd. for C_26_H_21_N_5_O_3_S (483.55), C, 64.58; H, 4.38; N, 14.48. Found: C, 64.38; H, 4.26; N, 14.19%.

##### Synthesis of compounds **7a**,**b**

These two compounds were prepared by the reaction of compound **1** (0.358 g, 1 mmol) with acetylacetone or ethylacetoacetate (1 mmol) in acetic acid (10 mL) in presence of ammonium acetate (0.20 g). The mixture was then heated under reflux to boiling for 3 h. The precipitate was collected by hot filtration and washed with ethanol to afford the desired product **7a**,**b**, respectively.

##### Ethyl 5-(5-acetyl-6-methylpyridin-2-yl)-4-methyl-2-(phenylamino)thiophene-3-carboxylate (**7a**)

Reddish brown powder, Yield (42%); mp 157–159 °C. IR (KBr, cm^−1^) ν_max_ = 3450 (NH), 1705 (C=O), 1654 (C=O), 1587 (C=N). ^1^H-NMR (600 MHz,CDCl_3_) *δ* (ppm): 1.42 (t, 3H, *J* = 6.1 Hz, CH_3_CH_2_), 2.58 (s, 3H, CH_3_), 2.64 (s, 3H, CH_3_CO), 2.76 (s, 3H, CH_3_-pyridine), 4.35 (q, 2H, *J* = 6.1 Hz, CH_3_CH_2_), 7.01–7.42 (m, 5H, Ar–H), 7.98 (d, 1H, *J* = 8.4 Hz, CH-pyridine), 10.49 (s, 1H, NH). MS (EIMS) *m/z*: 394 (M^+^, 100). Anal. Calcd. for C_22_H_22_N_2_O_3_S (394.49): C, 66.98; H, 5.62; N, 7.10. Found: C, 66.79; H, 5.50; N, 6.97%.

##### 6-4-Ethoxycarbonyl-3-methyl-5-phenylamino-thiophen-2-yl)-2-methyl-nicotinic acid ethyl ester (**7b**)

Bright orange needles. Yield (62%); mp 112–114 °C. IR (KBr, cm^−1^) ν_max_ = 3451 (NH), 1715 (C=O), 1656 (C=O), 1583 (C=N). ^1^H-NMR (600 MHz, CDCl_3_) *δ* (ppm): 1.39 (t, 3H, *J* = 6.1 Hz, CH_2_CH_3_), 1.43 (t, 3H, *J* = 6.1 Hz, CH_2_CH_3_), 2.46 (s, 3H, CH_3_), 2.75 (s, 3H, CH_3_), 4.36 (q, 2H, *J* = 6.1 Hz, CH_2_CH_3_), 4.37 (q, 2H, *J* = 6.1 Hz, CH_2_CH_3_), 7.15–7.42 (m, 6H, Ar–H), 8.18 (d, 1H, *J* = 8.4 Hz), 10.62 (s, 1H, NH–Ph). ^13^C-NMR (150 MHz, CDCl_3_) *δ* (ppm): 14.3, 14.4 (CH_2_ CH_3_), 16.8 (CH_3_), 30.3 (CH_3_-pyridine), 60.2, 60.7 (CH_2_CH_3_), 109.5, 110.0, 119.7, 120.3, 123.7, 124.7, 129.7, 138.8, 139.7, 146.0, 155.0, 161.6, 162.9 (Ar–C), 166.5, 167.0 (C=O). MS (EIMS) *m/z*: 425 (M^+^+1, 25), 259 (90), 167 (40), 139 (100), 97 (60), 43 (85). Anal. Calcd. for C_23_H_24_N_2_O_4_S (424.51):C, 65.07; H, 5.70; N, 6.60. Found: C, 64.89; H, 5.56; N, 6.48%.

##### Ethyl 5-(6-(4-(ethoxycarbonyl)-3-methyl-5-(phenylamino)thiophen-2-yl)nicotinoyl)-4-methyl-2-(phenylamino)thiophene-3-carboxylate (**8**)

This compound was prepared according to the following procedure: To a solution of compound **1** (0.358 g, 1 mmol) in glacial acetic acid (10 mL) in a 100 mL-flask with a condenser was added ammonium acetate (0.5 g). The mixture was heated to boiling for 5 h. The solid was filtered while hot to afford the desired product as reddish brown powder. Yield (54%); mp 108–110 °C. IR (KBr, cm^−1^) ν_max_ = 3451 (NH), 1706 (C=O), 1658 (C=O), 1593 (C=N). ^1^H-NMR (600 MHz, CDCl_3_) *δ* (ppm): 1.41 (t, 3H, *J* = 6.1 Hz, CH_2_CH_3_), 1.42 (t, 3H, *J* = 6.1 Hz, CH_2_CH_3_), 2.45 (s, 3H, CH_3_), 2.58 (s, 3H, CH_3_), 4.36 (q, 2H, *J* = 6.1 Hz, CH_2_CH_3_), 4.39 (q, 2H, *J* = 6.1 Hz, CH_2_CH_3_), 7.12–7.67 (m, 5H, Ar–H), 8.18 (d, 1H), 8.36 (d, 1H), 9.01 (s, 1H), 10.75 (s, 1H, NH–Ph), 10.78 (s, 1H, NH–Ph). ^13^C-NMR (125 MHz, CDCl_3_) *δ* (ppm): 14.4, 14.4 (CH_3_CH_2_), 18.3, 18.4 (CH_3_), 60.8, 61.0 (CH_3_ CH_2_), 110.3, 110.6, 120.5, 120.6, 124.6, 124.9, 125.4, 129.6, 129.7, 129.9, 130.0, 139.3, 139.4, 139.5, 141.1, 142.0, 147.4, 149.0, 164.4, 165.0, 166.9 (Ar–C), 166.9, 167.0 (C=O) for ester, 187.5 (C=O). MS (EIMS) *m/z*: 625 (M^+^ , 5), 321 (100), 292 (20), 122 (15), 126 (20), 83 (35). Anal. Calcd. for C_34_H_31_N_3_O_5_S_2_ (625.76); C, 65.26; H, 4.99; N, 6.72; Found: C, 65.09; H, 4.76; N, 6.57%.

##### Ethyl 4-methyl-2-(phenylamino)-5-(4,4,4-triethoxybut-2-enoyl)thiophene-3-carboxylate (**9**)

Fusion of enaminone **1** with triethylorthoformate in the presence of ZnCl_2_ gave compound **9**. Deep brown powder. Yield (55%); mp 210–212 °C. IR (KBr, cm^−1^) ν_max_ = 3450 (NH), 1705, 1680 (2C=O),1591 (C=O). ^1^H-NMR (600 MHz, CDCl_3_) *δ* (ppm): 1.39 (t, 9H, *J* = 6.1 Hz, 3CH_2_CH_3_), 1.40 (t, 3H, *J* = 6.1 Hz, CH_2_CH_3_), 2.17 (s, 3H, CH_3_), 4.35 (q, 6H, *J* = 6.1 Hz, 3CH_2_CH_3_), 4.36 (q, 2H, *J* = 6.1 Hz, CH_2_CH_3_), 5.39 (d, 1H, *J* = 12.1 Hz, CH=CH), 7.10–7.51 (m, 5H, Ar–H), 7.69 (d, 1H, *J* = 12.1 Hz, CH=CH), 10.51 (s, 1H, NH–Ph). ^13^C-NMR (125 MHz, CDCl_3_) *δ* (ppm): 14.4 (CH_2_CH_3_), 16.1 (CH_2_CH_3_), 16.9 (CH_3_), 57.6 (OCH_2_CH_3_), 60.3 (CH_2_CH_3_), 95.2, 153.0 (CH=CH), 109.7, 113.1, 119.7, 122.4, 123.8, 129.5, 140.3, 141.5, 160.7 (Ar–C), 167.2, 182.3 (C=O). MS (EIMS) *m/z*: 461 (M^+^, 6), 446 (30), 306 (100), 260 (40), 232 (70), 189 (65), 174 (60), 148 (52), 136 (55), 91 (80). Anal. Calcd. for C_24_H_31_NO_6_S (461.57); C, 62.45; H, 6.77; N, 3.03. Found: C, 62.29; H, 6.54; N, 3.12%.

##### Ethyl 5-(3-(hydroxyamino)acryloyl)-4-methyl-2-(phenylamino)thiophene-3-carboxylate (**10**)

This compound was prepared by addition of hydroxylamine hydrochloride (0.07 g, 1 mmol) to a solution of compound **1** (0.358 g, 1 mmol) in absolute ethanol (15 mL), in presence of anhydrous potassium carbonate (0.14 g, 1 mmol). The mixture was heated to boiling under reflux for 4 h. The solid product was filtered while hot and washed with ethanol to afford the desired product as bright brown powder. Yield (48%); mp > 300 °C. IR (KBr, cm^−1^) ν_max_ = 3427 (OH, NH), 1705 (ester C=O), 1655 (C=O). ^1^H-NMR (600 MHz, DMSO-*d*_*6*_) *δ* (ppm): 1.42 (t, 3H, *J* = 6.1 Hz, CH_2_CH_3_), 2.59 (s, 3H, CH_3_), 4.38 (q, 2H, *J* = 6.1 Hz, CH_2_CH_3_), 6.22 (d, 1H, *J* = 12.1 Hz, CH=CH), 7.08 (s, 1H, NH), 7.12–7.41 (m, 5H, Ar–H), 7.43 (s, 1H, NH–Ph), 8.24 (d, 1H, *J* = 12.1 Hz, CH=CH), 10.44 (s, 1H, OH). MS (EIMS) *m/z*: 346 (M^+^, 81), 287 (20), 255 (15), 228 (75), 195 (15), 169 (30), 113 (30), 91 (100). Anal. Calcd. for C_17_H_18_N_2_O_4_S (346.40): C, 58.95; H, 5.24; N, 8.09. Found: C, 58.76; H, 5.08; N, 7.94%.

##### 3.9. Ethyl 5-(3-((4-chlorophenyl)amino)acryloyl)-4-methyl-2-(phenylamino)thiophene-3-carboxylate(**11a**)

This compound was prepared as a yellow powder by addition of *p*-chloroaniline (0.127 g, 1 mmol) to a solution of **1** in absolute ethanol (15 mL) and the mixture was heated under reflux for 6 h. The product was filtered while hot to afford compound **11a**. Yield (40%); mp 110–112 °C. IR (KBr, cm^−1^) ν_max_ = 3450 (NH), 1645 (C=O), 1624 (C=O). ^1^H-NMR (600 MHz, DMSO-*d*_*6*_) *δ* (ppm): 1.32 (t, 3H, *J* = 6.1 Hz, CH_2_CH_3_), 2.62 (s, 3H, CH_3_), 4.29 (q, 2H, *J* = 6.1 Hz, CH_2_CH_3_), 5.30 (d, 1H, *J* = 12.1 Hz, HC=CH),5.32 (d, 1H, *J* = 12.1 Hz, HC=CH), 7.15–7.59 (m, 9H, Ar–H), 10.03 (s, 1H, NH–Ph) 10.14 (s, 1H, NH–Ph). ^13^C-NMR (150 MHz, DMSO-*d*_*6*_) *δ* (ppm): 14.7 (CH_2_CH_3_), 16.9 (CH_3_), 60.7 (CH_2_CH_3_), 94.3 (H^α^C=), 109.8, 120.5, 121, 123.4, 124.3, 124.8, 130.2, 130.2, 139.6, 139.8, 140.9, 160.1 (Ar–C), 153.7 (=^β^CH), 166.4 (O–C=O), 180.6 (C=O). MS (EIMS) *m/z*: 440 (M^+^, 79), 314 (100%). Anal. Calcd. for C_23_H_21_ClN_2_O_3_S (440.94) C, 62.65; H, 4.80; N, 6.35. Found: C, 62.43; H, 4.67; N, 6.19%.

##### Ethyl 4-methyl-2-(phenylamino)-5-(3-(p-tolylamino)acryloyl)thiophene-3-carboxylate (**11b**)

This compound was prepared as a deep grey powder by following the same procedure used to prepare compound **11a** and by using *m*-anisidine (0.123 g, 1 mmol) instead of *p*-chloroaniline. Yield(74%); mp 179–180 °C.IR (KBr, cm^−1^) ν_max_ = 3419, 3240 (NH), 1705 (C=O), 1659 (C=O). ^1^H-NMR (600 MHz, CDCl_3_) *δ* (ppm): 1.41 (t, 3H, *J* = 6 Hz, CH_2_CH_3_), 2.46 (s, 3H, CH_3_), 2.73 (s, 3H, CH_3_), 4.28 (q, 2H, *J* = 6.0 Hz, CH_2_CH_3_), 6.86 (d, 1H, *J* = 9.0 Hz, =^β^CH), 5.32 (d, 1H, *J* = 12.0 Hz, ^α^CH), 7.07–7.43 (m, 9H, Ar–H), 10.59, 10.62 (2 s, 2H, NH). Anal. Calcd. for C_24_H_24_N_2_O_3_S (420.53) C, 68.55; H, 5.75; N, 6.66. Found: C, 68.28; H, 5.56; N, 6.52%.

##### Agar diffusion medium

All compounds were screened in vitro for their antimicrobial activity by using the agar diffusion method [29]. A suspension of the organisms was added to sterile nutrient agar media at 45 °C and the mixture was transferred to sterile Petri dishes and allowed to solidify. Holes of 6 mm in diameter were made using a cork borer. The samples of the test compounds as well as reference drugs were dissolved in DMSO to give a solution of 5 mg mL^−1^. The amount tested compounds or reference drugs was 100 µL. Dimethylsulfoxide (DMSO) was used as a negative control. The plates were left for 1 h at room temperature as a period of pre-incubation diffusion to minimize the effects of variation in time between the applications of the different solutions. The plates were then incubated at 37 °C for 24 h and observed for antimicrobial activity. The diameters of inhibition zone were measured and compared with that of the reference drug. The observed inhibition zones were measured in millimeter beyond well diameter. Also, the percentage value of inhibition zones compared to reference drugs were recorded (Tables 1, 2).

**Table 2:** Antifungal activity of synthesized compound (zone of inhibition in diameter in mm)

Tested microorganisms comp. no	Fungi Inhibition zone diameter in mm and (%) value			
	*Aspergillus fumigates*	*Syncephalastrum racemosum*	*Geotricum candidum*	*Candida albicans*
St.	Amphotericin B			
	23.7 ± 0.1	19.7 ± 0.2	28.7 ± 0.2	25.4 ± 0.1
**2**	15.7 ± 0.33 (66.2%)	13.8 ± 0.25 (70.1%)	18.3 ± 0.34 (64.8%)	15.2 ± 0.53 (59.8%)
**3**	18.7 ± 0.36 (78.9%)	16.9 ± 0.27 (85.8%)	13.4 ± 0.65 (46.7%)	10.9 ± 0.23 (42.9%)
**4**	16.3 ± 0.53 (68.8%)	13.4 ± 0.49 (68.0%)	15.9 ± 0.71 (55.4%)	17.3 ± 0.62 (68.1%)
**5**	15.9 ± 0.62 (67.1%)	18.9 ± 0.58 (95.9%)	19.1 ± 0.54 (66.6%)	15.8 ± 0.38 (62.2%)
**6**	18.2 ± 0.57 (76.8%)	17.4 ± 0.6 (88.3%)	17.8 ± 0.72 (62.0%)	12.9 ± 0.37 (50.8%)
**7a**	12.3 ± 0.39 (51.9%)	17.2 ± 0.16 (87.3%)	24.6 ± 0.58 (85.7%)	12.7 ± 0.38 (50.0%)
**7b**	23.7 ± 0.1 (100%)	19.7 ± 0.2 (100%)	28.7 ± 0.2 (100%)	25.4 ± 0.1 (100%)
**8**	23.7 ± 0.1 (100%)	19.7 ± 0.2 (100%)	28.7 ± 0.2 (100%)	25.4 ± 0.1 (100%)
**9**	14.9 ± 0.61 (62.9%)	13.7 ± 0.42 (69.5%)	15.9 ± 0.38 (55.4%)	14.7 ± 0.52 (57.9%)
**10**	17.3 ± 0.49 (73.0%)	13.3 ± 0.39 (67.5%)	14.6 ± 0.52 (50.9%)	15.1 ± 0.48 (59.4%)
**11a**	13.6 ± 0.25 (57.4%)	11.7 ± 0.34 (59.4%)	16.5 ± 0.58 (57.5%)	13.4 ± 0.45 (52.8%)
